# Are upright lateral cervical radiographs in the obtunded trauma patient useful? A retrospective study

**DOI:** 10.1186/1749-7922-2-4

**Published:** 2007-02-08

**Authors:** Craig H Rabb, Jeffrey L Johnson, David VanSickle, Kathryn Beauchamp, Gene Bolles, Ernest E Moore

**Affiliations:** 1 Department of Neurosurgery, Denver Health Medical Center, University of Colorado School of Medicine, Denver, CO, USA; 2 Department of Surgery, Denver Health Medical Center, University of Colorado School of Medicine, Denver, CO, USA

## Abstract

**Background:**

The best method for radiographic "clearance" of the cervical spine in obtunded patients prior to removal of cervical immobilization devices remains debated. Dynamic radiographs or MRI are thought to demonstrate unstable injuries, but can be expensive and cumbersome to obtain. An upright lateral cervical radiograph (ULCR) was performed in selected patients to investigate whether this study could provide this same information, to enable removal of cervical immobilization devices in the multiple trauma patient.

**Methods:**

We retrospectively reviewed our experience with ULCR in 683 blunt trauma victims who presented over a 3-year period, with either a Glasgow Coma Score <13 or who were intubated at the time of presentation.

**Results:**

ULCR was performed in 163 patients. Seven patients had studies interpreted to be abnormal, of which six were also abnormal, by either CT or MRI. The seventh patient's only abnormality was soft tissue swelling; MRI was otherwise normal. Six patients had ULCR interpreted as normal, but had abnormalities on either CT or MRI. None of the missed injuries required surgical stabilization, although one had a vertebral artery injury demonstrated on subsequent angiography. ULCR had an apparent sensitivity of 45.5% and specificity of 71.4%.

**Conclusion:**

ULCR are inferior to both CT and MRI in the detection of cervical injury in patients with normal plain radiographs. We therefore cannot recommend the use of ULCR in the obtunded trauma patient.

## Background

There is considerable debate regarding "clearance of the cervical spine" in trauma patients who are uncooperative or unable to give a reliable history [1]. Much of the debate centers around what is the minimum radiographic workup necessary prior to removing cervical immobilization devices [2]. In particular, detection of instability in the absence of bony fractures is of concern to all who treat the trauma patient. Most of the attention in the literature has focused on the use of either flexion-extension radiographs or MRI for this purpose. There has been concern about the potential of the former to cause neurologic injury. Conversely, some have argued that MRI must be performed within 48 hours to be useful, while others have challenged its specificity [3-5]. The need to rapidly remove immobilization devices is additionally vital to reduce the risk of pressure-related skin breakdown [6]. These shortcomings have led to the search for other studies that might identify patients with instability, when plain radiographs and CT scans are normal.

At this institution, between 2002 and 2005, a protocol was established for the evaluation of the cervical spine in the trauma patient that included an upright lateral cervical radiograph (ULCR) in patients who remained unable to reliably answer questions about neck pain, had no apparent neurologic deficits, and normal plain cervical radiographs. The rationale for the use of the ULCR was similar to the protocol later described by Griffen and co-workers [7]. Specifically, the theory that elevating the patient to 90°, while still in the collar for the exam, will provide a "stressed" exam which may uncover an unstable injury. Griffen has speculated that, as patients are moved by the nursing staff for routine nursing care, unstable injuries may be revealed when the upright film is performed. We sought to review this experience to determine whether this exam provided a reliable basis for determining the safety of removing cervical immobilization.

## Methods

Approval from the Colorado Multiple Institutional Review Board was obtained prior to initiation of this study (COMIRB #04-1118). The Trauma Registry at Denver Health Medical Center, an academic regional Level I Trauma Center, was queried to identify all those patients who presented to our institution between April 1, 2002 thru March 31, 2005 with a history trauma, who had a Glasgow Coma Score of 12 or less, or who were intubated prior to or shortly after arrival to DHMC. These criteria were selected so as to find the highest yield of patients having undergone ULCR as part of their workup to identify cervical instability. Patients with a diagnosis of penetrating trauma were excluded, as were patients under the age of 18. The records were further reviewed to determine if there were any injuries to the cervical spine noted on any other radiographic study performed in these patients, for comparison. Results of any angiographic studies performed to evaluate for any blunt carotid or vertebral artery injuries (BCVI) were also reviewed; the Denver BCVI Classification scheme was employed [8].

## Results

A total of 683 patients were identified with a history of blunt trauma, who were either intubated or had a Glasgow Coma Score of 12 or less. Mean Injury Severity Score (ISS) was 29.3. There were 550 men and 133 women. Of these, 163 had ULCR performed. Four of the 163 were read as inadequate or equivocal. Two of these four patients with inadequate ULCR had a CT scan that demonstrated a C7 transverse process fracture. One patient's examination was normal thru C6, but had a C6 pedicle fracture and C6/7 facet fracture determined by both CT and MRI. Interestingly, the plain radiographs were interpreted as normal. That patient had a normal vertebral and carotid angiogram, and underwent surgical stabilization. The last patient with equivocal ULCR had a normal MRI and the orthosis was removed. In the remaining 159 patients, 11 injuries were confirmed by either CT or MRI, for a prevalence of 6.9%.

Seven patients of the 159 remaining patients (4.4%) had ULCR that were interpreted as abnormal (Table 1). One of these was interpreted as having soft tissue swelling, but had a normal MRI. One was interpreted as having subluxation at C3/4, but had a normal MRI. Five had abnormalities that were also noted on CT and MRI. Assuming CT or MRI to be the gold standard, 5 of 7 patients with an abnormal ULCR proved to have an actual injury, for a specificity of 71.4%.

**Table 1:** Upright Lateral Cervical Radiographs (ULCR) Interpreted As Abnormal

**ULCR**	**Plain Radiographs**	**CT**	**MRI**	**Angiogram**
Soft tissue swelling	Normal		Normal	
Atlantoaxial joint injury	Atlantoaxial joint injury		Atlantoaxial joint injury	
C3/4 4 mm subluxation	Normal		Normal	
C7 Body fracture		C7 body fracture	C7 body fracture	Grade II right vertebral artery injury
C6/7 Facet fracture	C7 body fracture	C7 body fracture	C7 body fracture	Normal
C7 Facet fracture	Normal thru C7	C7 Facet fracture		Normal
C 5/6,6/7 1–2 mm subluxation		C5 Spinous process, lamina fractures		

Review of these data was also noteworthy for identifying six patients (3.8%) whose ULCR were interpreted as normal, but whose supplemental imaging (CT and MRI) detected abnormalities (Table 2). Overall, 6 of 11 cervical injuries identified from the population of 159 patients were missed by the ULCR, yielding apparent sensitivity of 45.5% for ULCR. One patient had a C7 transverse process fracture per MRI; angiography was normal. Two patients had C2 lateral mass fractures by CT. Both had normal angiograms. One patient had a C7 facet fracture, identified by both MRI and CT. This was treated conservatively, and patient had a normal angiogram. Two patients had more serious injuries. One had a C1/2 facet injury, which was seen on CT and MRI. Angiography was normal. The most serious injury missed by the ULCR was a C4 pedicle with C4/5 facet injury. This patient had a Grade I vertebral artery injury at the level of C4/5. This patient also had significant traumatic brain injury, which resulted in his spine injury being treated conservatively.

**Table 2:** Upright Lateral Cervical Radiographs (ULCR) Interpreted As Normal, but with Abnormalities on other Imaging Modalities

***ULCR***	**Plain Radiographs**	**CT**	**MRI**	**Angiogram**
Normal	Normal		C7/T1Transverse, process fractures	Normal
Normal	Normal	C4 Pedicle fracture	C4/5 Edema, C5/6 facet injury	Grade I R vertebral artery injury
Normal	Normal	C1/2 facet joint injury	C1/2 facet joint injury	Normal
Normal	Normal	C2 lateral mass fracture		Normal
Normal	Normal	C2 lateral mass fracture		
Normal	Normal	C7 Facet fracture	C7 facet fracture	Normal

## Discussion

The ideal method for excluding unstable cervical spine injuries in blunt trauma victims has remained elusive. A recent rigorous review of the literature has concluded that, for patients with no neck pain, no radiographic workup is necessary [9]. Therefore, the primary area of controversy centers around those patients unable to give an adequate history. For trauma victims, this group is typically patients with altered mental status, or those who remain intubated. The ability to rapidly identify patients with no cervical spine injury is especially important, as it is well-known that prolonged placement of a cervical immobilization device can lead to pressure ulcers [6]. In addition to the risk of pressure ulcers, continued cervical immobilization may impair overall mobilization of the critically ill patient, or otherwise limit treatment options for patients with significant pulmonary compromise.

Bony injuries of the spine are readily identified by contemporary CT imaging [1,10-15]. Consequently, identification of unstable injuries in the absence of bony fractures is important. For such patients, either MRI or dynamic flexion-extension studies have been proposed as the ideal radiographic examination.

Flexion-extension radiographs have been employed as a gold standard for the identification of spinal instability [16-19]. However, there are many drawbacks to that test. It is labor-intensive, fraught with the potential for insufficient or inadequate exams, and highly impractical in critically injured patients [20]. Many technicians are reluctant to assist with performance of this test for fear of causing neurologic deficits, even though numerous authors have documented safety in this population [20-23]. In addition, some authors have expressed concern for problems with both sensitivity and specificity of this study [4,5]. Moreover, it has been suggested that these studies are better suited to being performed days to weeks post-injury, to reduce any confounding effect that may result from muscle spasm [24]. The latter may further increase the false-negative rate by limiting motion. As a result of these concerns, MRI has been promoted as an alternative when supplementary imaging has been desired.

It is widely recognized that MRI is better suited than CT for visualization of the soft-tissues [25,26]. D'Alise et al, argued in favor of MRI prior to CT, citing concerns about inability to visualize soft-tissue changes in the normally-aligned spine that is imaged by CT [23]. Conversely, Brohi and co-workers maintain that unstable disc or ligamentous injury may be detected indirectly by CT, through evaluation of malalignment or malrotation [27]. Indeed, we have seen subtle abnormalities such as facet joint widening, which led to confirmatory MRI studies. Not surprisingly, MRI has been demonstrated to be very accurate for identifying traumatic disc herniation [13,25,28]. The impact of the presence of disc herniations on the decision as to whether or not to remove an immobilization device remains unclear.

A recent effort to delineate evidence-based guidelines for the exclusion of cervical instability recommended that MRI be obtained within 48 hours in order to be valid [3]. Horn, et al recently examined the use of MRI for the purpose of identifying posttraumatic instability [18]. In their series of 70 patients whose only positive findings were soft tissue injuries on MRI, none were noted to have instability when flexion-extension radiographs were performed. This finding raises the question as to the value of MRI for identifying cervical instability, as it appears to have a high false-positive rate. Schuster and colleagues conducted a prospective comparison of MRI and CT imaging in patients presenting with blunt trauma and concluded that, for patients without obvious motor weakness, MRI was unnecessary for clearance of the cervical spine [29]. Thus, the best diagnostic regimen for evaluating occult instability remains unclear [30].

At our institution, we attempt to clarify the presence or absence of cervical injuries rapidly, so that immobilization devices may be removed quickly, to reduce the potential for skin breakdown and to assist with early mobilization of the patient. The ULCR was proposed as a potential alternative to MRI or dynamic radiographs, in hope of providing a rapid, inexpensive means of identifying cervical instability in trauma patients unable to reliably give a history of pain, in whom radiographs were otherwise normal. Given the lack of reliable information regarding the value of this exam, we sought to analyze this experience to determine if this test has value in the trauma patient.

Identifying 163 patients undergoing ULCR out of 683 possible patients would seem to indicate a small number of those potentially eligible for the test. The retrospective methodology employed is reliant on data that were recorded at presentation. Many of the patients with these criteria at admission had a cervical injury excluded through other means; once patients were extubated, standard radiographs plus physical exam was considered adequate. Other patients had obvious cervical injuries at admission and had other radiographic studies, and some patients died from their injuries, accounting for the minority of potentially eligible patients who underwent ULCR.

Of the injuries missed by the ULCR, none required surgical stabilization. However, these injuries cannot be regarded as insignificant. In addition to the frequent need for maintaining a cervical immobilization device in such patients, at our institution, angiography is now routinely performed for cervical spine fractures involving subluxation, extension into the foramen transversarium, or C1 to C3 fractures. This type of fracture pattern has been demonstrated to be associated with vertebral artery injuries [31]. Indeed, vertebral artery injuries were noted in this series, further supporting an aggressive approach to identifying fractures, even if they are considered stable and only require an orthosis for treatment (Table 1, Table 2).

The retrospective nature of this study, combined with the heterogeneous patient population make interpretation of these data open to question. Not every patient received a uniform workup (CT plus MRI). Thus, the actual sensitivity of ULCR may be even lower. Regardless, given the facts that a) no injuries were detected that were not otherwise detected also by CT or MRI, and b) several injuries were missed, we submit that this radiographic examination is of little value. We have, therefore, abandoned the use of this test and cannot recommend it.

## Conclusion

Recognizing the limitations of retrospective studies, we conclude on the basis of this institution's experience that upright cervical radiographs are unreliable in the assessment of cervical instability in the blunt trauma patient.
